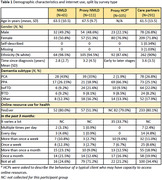# Digital access for non‐memory‐led and inherited dementias: How to optimise blended digital/human interventions

**DOI:** 10.1002/alz.090931

**Published:** 2025-01-09

**Authors:** Emilie V Brotherhood, Celine El Baou, Caroline Fearn, Oliver S Hayes, Nikki Zimmermann, Anastasia Tsipa, Joshua Stott

**Affiliations:** ^1^ Dementia Research Centre, UCL Queen Square Institute of Neurology, University College London, London United Kingdom; ^2^ Department of Clinical, Educational, and Health Psychology, Division of Psychology and Language Sciences, University College London, London United Kingdom

## Abstract

**Background:**

How people affected by non‐memory‐led and inherited dementias (NMLDs) interact with online health resources is poorly understood. We conducted the world’s largest survey exploring ‘digital access in non‐memory‐led dementias’ to learn directly from people with NMLD, their care partners and NMLD Healthcare Professionals (HCPs) about the NMLD experience interacting with web‐based health resources.

**Method:**

Four surveys [for individuals diagnosed with NMLD, care partners, care partner proxy for person with NMLD, HCP proxy] were co‐developed with people with NMLD experience. Surveys contained standardised scales and open‐ended short‐form questions relating to: behavioural intention to use web‐based resources (Venkatesh, 2012), attitudes towards web‐based resources, gerontechnology‐related anxiety, control beliefs, health and quality of life (Chen & Lou, 2020) digital health literacy (Nelson, 2022) and web‐related privacy/security concerns (Hong & Thong, 2013). The survey was administered either: online via Qualtrics, or in hard copy via post/in‐person data collection at Rare Dementia Support (RDS) meetings.

**Result:**

Initial inspection of the data indicates 572 responses were collected from 481 individuals. Responses were received from: people with NMLD (N = 65; years since diagnosis mean±sd = 2.81 ± 2.56 years); care partners (N = 291); care partners providing proxy NMLD responses (N = 111); and NMLD HCPs providing perspectives from their experience of service users’ interactions with digital health resources (N = 105). From preliminary inspection of partial data (see Table 1) the proportion of positive responses to the question ‘Have you (proxy: person with NMLD) ever used web‐based resources (e.g., Zoom, websites) for your health and psychological wellbeing?’ is similarly reported between people living with NMLD (80.0%) and proxy HCP responses (79.0%), but a lower proportion reported in proxy caregiver responses (51.3%). In the proxy NMLD responses, the proxy estimation of people with NMLD accessing online resources in the past 3 months is low (71.2% not accessing any web‐based health resources three months prior to survey completion).

**Conclusion:**

Initial analyses indicate this dataset will provide insight for practitioners, clinicians and research academics in the development of online health resources for people affected by NMLD.